# Editorial: Challenges in dementia care: a global perspective

**DOI:** 10.3389/fpsyt.2024.1450368

**Published:** 2024-07-08

**Authors:** Carlo Custodero

**Affiliations:** Clinica Medica “A. Murri”, Department of Precision and Regenerative Medicine and Ionian Area (DiMePrev-J), University of Bari Aldo Moro, Bari, Italy

**Keywords:** cognitive decline, behavioral and psychological symptoms of dementia, caregiver burden, mortality, environmental factors

After decades of research efforts, dementia care is still an open challenge. Some promising results come from disease modifying therapies in dementia due Alzheimer’s disease (AD) as the anti-amyloid antibodies lecanemab and donanemab (1). However the association between amyloid reduction strategies and a clinically meaningful improvement of cognitive outcomes remains questionable (1). Probably one of the few certainties is still the importance of the dementia prevention strategies (2). Indeed, an increasing knowledge and perception of the problem among people and healthcare systems, has led to observe in the last few years a promising trend toward reduction of incident dementia cases (2). However, nowadays there are around 57 million people worldwide living with dementia and the great burden of disease is attributable to the neurobehavioral symptoms (3). Such manifestations occur in most of individuals with dementia at some point of the disease history, increasing the caregiver burden and adversely affecting the quality of life for the patient and caregiver (4).

Our Research Topic aims to better explore some challenging epidemiological and clinical aspects among people with dementia across different settings (e.g. community, nursing home) and countries. The Research Topic includes five original articles examining the risk factors associated with mortality and further worsening of cognition among subjects with dementia and the predictors of Behavioral and Psychological Symptoms of Dementia (BPSD) severity.

Heng et al. explored the difference in mortality rate attributable to dementia across different areas of a Chinese province showing a great impact of socioeconomical and environmental factors (i.e., poor economic status, insufficient health resources, and worse pollution) in determining higher risk of dementia mortality. This data underlined the importance to improve and broaden prevention strategies in low- and middle-income countries.

Another aspect, that emerged from the work of Sánchez-Valdeón et al., are the long-term indirect effects of COVID-19 pandemic among older adults living with dementia. The study highlighted the effects on cognition of the lockdown period in the subjects referred to the adult day service centers. The interruption during that period of non-pharmacological intervention, like cognitive stimulation and occupational therapy aimed to temporarily delay disability and relieve psychological distress, led to significant worsening of cognitive function compared to the pre-pandemic period observing a Mini-Mental State Examination (MMSE) reduction of more than 10-point/year in 33.8% of participants during lockdown versus 5.5% earlier.

In a cross-sectional study, Lu et al. demonstrated an higher severity of the BPSD in subjects with moderate-to-severe dementia living in the community compared to those in nursing home leading also to almost three times higher caregiver burden. Family caregiving emerged as an independent risk factor for clinically significant BPSD and moderate to severe caregiver distress. The article by Leng et al. carried out among 1012 dementia patient-carer dyads identified nine core care problems perceived by caregivers, and the most reported were: decline in autonomy, verbal and nonverbal aggression and loss of activities of daily living. These problem clusters were strictly and independently related to other important factors as marital status, years of dementia diagnosis, number of dementia medication type, and caregiver’s educational levels. Pivotal is also the environment where the patients with dementia live. Indeed, the experience reported by Ishimaru et al. suggested that some environmental triggers were associated with visual hallucination in patients with dementia with Lewy bodies. Therefore the recognition of these elements could help to promote environmental adjustments and avoid recurrencies.

In conclusion, the papers included in this Research Topic offer valuable insights into risk factors related to mortality and progression of cognitive impairment in subjects with dementia. Better understanding of such epidemiological aspects could help to improve dementia care in particular in low-middle income countries which already count the great prevalence of dementia cases. Moreover deeper knowledge on the predictors of BPSD severity and caregiver burden could allow to strengthen specific aspects in social assistance finally improving the quality of life of the patients and carers.

## Author contributions

CC: Writing – original draft.
